# Mortality differences by surgical volume among patients with stomach cancer: a threshold for a favorable volume-outcome relationship

**DOI:** 10.1186/s12957-017-1203-7

**Published:** 2017-07-17

**Authors:** Hyeok Choi, Seong-Yoon Yang, Hee-Seung Cho, Woorim Kim, Eun-Cheol Park, Kyu-Tae Han

**Affiliations:** 1 Premedical Courses, Yonsei University College of Medicine, Seoul, Republic of Korea; 2 Department of Public Health, Graduate School, Yonsei University, Seoul, Republic of Korea; 3 Institute of Health Services Research, Yonsei University, Seoul, Republic of Korea; 4 Department of Preventive Medicine, Yonsei University College of Medicine, Seoul, Republic of Korea; 5 Research and Analysis Team, National Health Insurance Service Ilsan Hospital, Goyang, Republic of Korea

**Keywords:** Stomach neoplasms, High-volume hospitals, Mortality, Cox proportional hazard models, Gastrectomy

## Abstract

**Background:**

Many studies have assessed the volume-outcome relationship in cancer patients, but most focused on better outcomes in higher volume groups rather than identifying a specific threshold that could assist in clinical decision-making for achieving the best outcomes. The current study suggests an optimal volume for achieving good outcome, as an extension of previous studies on the volume-outcome relationship in stomach cancer patients.

**Methods:**

We used National Health Insurance Service (NHIS) Sampling Cohort data during 2004–2013, comprising healthcare claims for 2550 patients with newly diagnosed stomach cancer. We conducted survival analyses adopting the Cox proportional hazard model to investigate the association of three threshold values for surgical volume of stomach cancer patients for cancer-specific mortality using the Youden index.

**Results:**

Overall, 17.10% of patients died due to cancer during the study period. The risk of mortality among patients who received surgical treatment gradually decreased with increasing surgical volume at the hospital, while the risk of mortality increased again in “high” surgical volume hospitals, resulting in a j-shaped curve (mid-low = hazard ratio (HR) 0.773, 95% confidence interval (CI) 0.608–0.983; mid-high = HR 0.541, 95% CI 0.372–0.788; high = HR 0.659, 95% CI 0.473–0.917; ref = low). These associations were especially significant in regions with unsubstantial surgical volumes and less severe cases.

**Conclusion:**

The optimal surgical volume threshold was about 727.3 surgical cases for stomach cancer per hospital over the 1-year study period in South Korea. However, such positive effects decreased after exceeding a certain volume of surgeries.

## Background

Cancer is the most common health issue in South Korea due to its aging population, in which the prevalence rate of chronic diseases has been increasing [1]. To reduce problems related to cancer, many studies have been conducted and treatment strategies, such as endoscopic procedures, surgery, chemotherapy, and radiotherapy that considers a patient’s condition according to each cancer stage, were developed [2, 3]. Hence, survival rates of cancer patients showed a rapid increase of about 25% over the recent decade, and cancer is increasingly being considered as a type of chronic disease that requires timely diagnosis and treatment rather than as an incurable disease [4]. Nevertheless, cancer incidence has generally increased, and it remains as the most frequent cause of death (age-standardized mortality rate 90.2 per 100,000; 27.6% of all deaths in 2012) [4]. Among all types of cancer, stomach cancer is the second most frequent and the third highest causes of death in South Korea (age-standardized incidence rate 39.9 per 100,000 in 2012; age-standardized mortality rate 11.2 per 100,000 in 2012) [4]. Given the high frequency and mortality of stomach cancer compared to other cancers, it is necessary to devise effective alternatives for managing South Korean stomach cancer patients.

Stomach cancer is usually treated by surgical treatment rather than by chemotherapy or radiotherapy [5]. Previous surgical studies proposed many theories and alternatives to achieve better surgical outcomes, and the volume-outcome relationship was described in most cases. This was because physicians or hospital personnel who treat more surgical cases can develop their skills, resulting in better outcomes, although an excessive volume could lead to negative outcomes [6]. Given that the number of cancer patients in early stages of the disease has gradually increased due to new medical technologies and screening methods, the number of patients who undergo surgical treatment has also increased [7–9]. Therefore, many studies focusing on the volume-outcome relationship for cancer patients have been published [10–12]. However, most of the previous studies focused on better outcomes in higher volume groups, rather than suggesting a specific volume threshold that can guide clinical decision-making to improve outcomes. In addition, only a few studies are available regarding the volume-outcome relationship for cancer patients in South Korea and other Asian countries. Therefore, we investigated the relationship between surgical volume for stomach cancer patients and mortality as an extension of previous studies on the volume-outcome relationship in cancer patients, using nationally representative data in South Korea. Our findings suggest an optimal volume for achieving good outcomes for stomach cancer patients, and the results may assist in the clinical decision-making and establishment of effective health policies.

## Methods

### Database and data collection

We used the National Health Insurance Service (NHIS) Sample Cohort data during 2002–2013 in this study. This data included a 1,025,340 population which was collected through a systematic sampling to extract a 2.2% nationally representative sample among 46,605,433 Koreans in 2002, and it included all healthcare claims that were filed during 2002 to 2013. To analyze the relationship of stomach cancer surgical volume for cancer-specific mortality, we only included patients with newly diagnosed stomach cancer (International Classification of Diseases (ICD)-10: C16) and undergoing gastrectomy as their first treatment since 2004. Finally, the data used in this study were from 2550 newly diagnosed stomach cancer patients who received surgical treatment from 2004 to 2013.

### Variables

The dependent variable used in this study was mortality of patients with newly diagnosed stomach cancer (ICD-10: C16). It was defined as cancer-specific mortality in patients with stomach cancer. We selected the first date of each patient’s hospital visit regardless of types of visits and followed the stomach cancer patient. Patient mortality in the study population was assumed to be the result of worsening status due to cancer.

The interesting variable in this study was surgical volume for stomach cancer patients at each hospital over a 1-year period. To identify the threshold for optimal surgical volume in a positive volume-outcome relationship, we analyzed the optimal cutoff value of surgical volumes to achieve an efficient volume-outcome relationship related to cancer-specific mortality, using the Youden index (sensitivity + specificity − 1) and the formula shown below. The Youden index is the maximum vertical distance between ROC and a diagonal line; it is the idea for maximizing the difference between true positive and false positive. The optimal cutoff refers to where the Youden index is at its highest value [13–15].

$$ \mathrm{Cutoff}\ \mathrm{volume}=\left\{ \log \left(\frac{p}{1- p}\right) - {\beta}_0\right\}\div {\beta}_1 $$

*P* = probability at maximizing the Youden index; *β*_0_ = intercept; *β*_1_ = coefficient of surgical volume.

Based on the cutoff volume, we categorized the surgical volume into two groups and then additionally calculated the cutoff values for each group. By estimation, we selected the following threshold values: low (<3.0), mid-low (3.0–16.0), mid-high (16.0–30.0), and high (>30.0).

We also adjusted other independent variables to analyze the association between the surgical volume in each hospital and the mortality of patients who underwent surgical treatment for stomach cancer. Other independent variables were sex, age, income level, types of insurance coverage, types of surgery, types of treatment, dissection of lymph node, Charlson Comorbidity Index (CCI), region, and types of medical institution. We divided the age groups by intervals of 10 years from 40 to 70 years to reflect the variety in patient outcomes according to aging, and below 30 years and above 70 years were merged, respectively. Types of insurance coverage in South Korean were defined into three groups as medical aid, National Health Insurance (NHI) employee insurance, or NHI self-employed insurance according to the National Health Insurance act. Beneficiaries of NHI employee insurance comprised of either workers or employers in workplaces and also included their family who live together. They pay about 7% of their average salary as premium, and the rates changed every year. Beneficiaries of NHI self-employed insurance were people for whom the criteria for the NHI employee did not apply. Their premium is accounted based on both their income which does not occur in the workplace and their property. Medical aid was applied to people with lower income compared to government-defined poverty level and people with a disability. They could be provided free medical care by government funds. By those criteria of NHI benefit, the type of insurance coverage could reflect the socioeconomic status of South Koreans. To reflect the severity of stomach cancer in patients, we included treatment variables. Types of surgery were classified into total and subtotal gastrectomy, as well as by whether lymph node dissection was included [16, 17]. Types of treatment were classified into surgery with chemotherapy and radiotherapy, surgery with chemotherapy, surgery with radiotherapy, and only surgery [18]. The CCI was calculated by weighting and scoring for other comorbid conditions besides cancer [19]. Hospital region was defined as hospitals that provided surgical treatment for patients with stomach cancer in capital regions (Seoul and Gyeonggi) and others [20].

### Statistical analysis

In this study, we analyzed the descriptive statistics such as frequency or percentages of the study population in a categorical variable at the baseline of study, and then we analyzed chi-square tests for categorical variables and cancer-specific mortality. In continuous variables, we showed the means and standard deviations as descriptive statistics and also analyzed *t* tests by cancer-specific mortality. For suggesting the differences of cancer-specific survival times by the surgical volume groups, we showed the Kaplan-Meier survival curves with log-rank tests. Finally, to suggest the association of surgical volume in each hospital for cancer-specific mortality, we analyzed survival analyses adopting the Cox proportional hazard model while adjusting other covariates. Sub-group analyses were also performed to investigate the differences in the association of surgical volume for cancer-specific mortality according to region and types of surgery or treatment. SAS statistical software version 9.4 (Cary, NC) was used in all analyses of this study.

## Results

There were 2550 patients with newly diagnosed stomach cancer during 2004 to 2013. Overall, 17.10% of patients died due to cancer over the study period. Stomach cancer patients who received surgical treatment at hospitals with higher surgical volumes had lower mortality. Patients with vulnerable socioeconomic status, such as old age or low finances, experienced higher mortality. With regard to severity indicators, patients who received total gastrectomy or complex therapy had higher mortality. In addition, patients who died from cancer had higher CCI scores (Table 1).

**Table 1:** General characteristics of patients with stomach cancer at baseline and distribution of those by cancer-specific mortality

Variables	Total		Died		Survived		*P* value
	*N*/mean	%/SD	*N*/mean	%/SD	*N*/mean	%/SD	
Sex							
Male	1739	68.20	314	18.06	1425	81.94	0.0598
Female	811	31.80	122	15.04	689	84.96	
Age (years)							
−39	110	4.31	14	12.73	96	87.27	<0.0001
40–49	379	14.86	48	12.66	331	87.34	
50–59	650	25.49	82	12.62	568	87.38	
60–69	768	30.12	137	17.84	631	82.16	
70+	643	25.22	155	24.11	488	75.89	
Income							
−40	716	28.08	127	17.74	589	82.26	0.6956
41–70	678	26.59	113	16.67	565	83.33	
71–90	692	27.14	124	17.92	568	82.08	
91+	464	18.20	72	15.52	392	84.48	
Types of insurance coverage							
Medical aid	87	3.41	23	26.44	64	73.56	0.0399
NHI, self-employed	915	35.88	162	17.70	753	82.30	
NHI, employed	1548	60.71	251	16.21	1297	83.79	
Year of surgery							
2004	238	9.33	70	29.41	168	70.59	<0.0001
2005	244	9.57	59	24.18	185	75.82	
2006	229	8.98	50	21.83	179	78.17	
2007	216	8.47	60	27.78	156	72.22	
2008	266	10.43	46	17.29	220	82.71	
2009	252	9.88	48	19.05	204	80.95	
2010	263	10.31	44	16.73	219	83.27	
2011	288	11.29	26	9.03	262	90.97	
2012	280	10.98	27	9.64	253	90.36	
2013	274	10.75	6	2.19	268	97.81	
Types of surgery							
Total gastrectomy	552	21.65	174	31.52	378	68.48	<0.0001
Subtotal gastrectomy	1998	78.35	262	13.11	1736	86.89	
Types of treatment							
With chemotherapy and radiotherapy	44	1.73	27	61.36	17	38.64	<0.0001
With chemotherapy	373	14.63	178	47.72	195	52.28	
With radiotherapy	9	0.35	7	77.78	2	22.22	
Only surgery	2124	83.29	224	10.55	1900	89.45	
Dissection of lymph node							
Yes	2531	99.25	431	17.03	2075	81.98	0.3081
No	44	1.73	5	11.36	39	88.64	
CCI	0.79	1.43	1.05	1.72	0.74	1.36	<0.0001
Hospital characteristics							
Hospital volume							
Low (*N* = 368)	436	17.10	59	13.53	377	86.47	<0.0001
Mid-low (*N* = 292)	357	14.00	40	11.20	317	88.80	
Mid-high (*N* = 27)	1273	49.92	227	17.83	1046	82.17	
High (*N* = 17)	484	18.98	110	22.73	374	77.27	
Region of hospital							
Capital area (*N* = 328)	951	37.29	150	15.77	801	84.23	0.1705
Others (*N* = 376)	905	35.49	286	31.60	1313	145.08	
Types of medical institution							
General hospital (*N* = 689)	2531	99.25	430	16.99	2101	83.01	0.0924
Hospital (*N* = 15)	19	0.75	6	31.58	13	68.42	
Total	2550	100.00	436	17.10	2114	82.90	

**Fig. 1:**
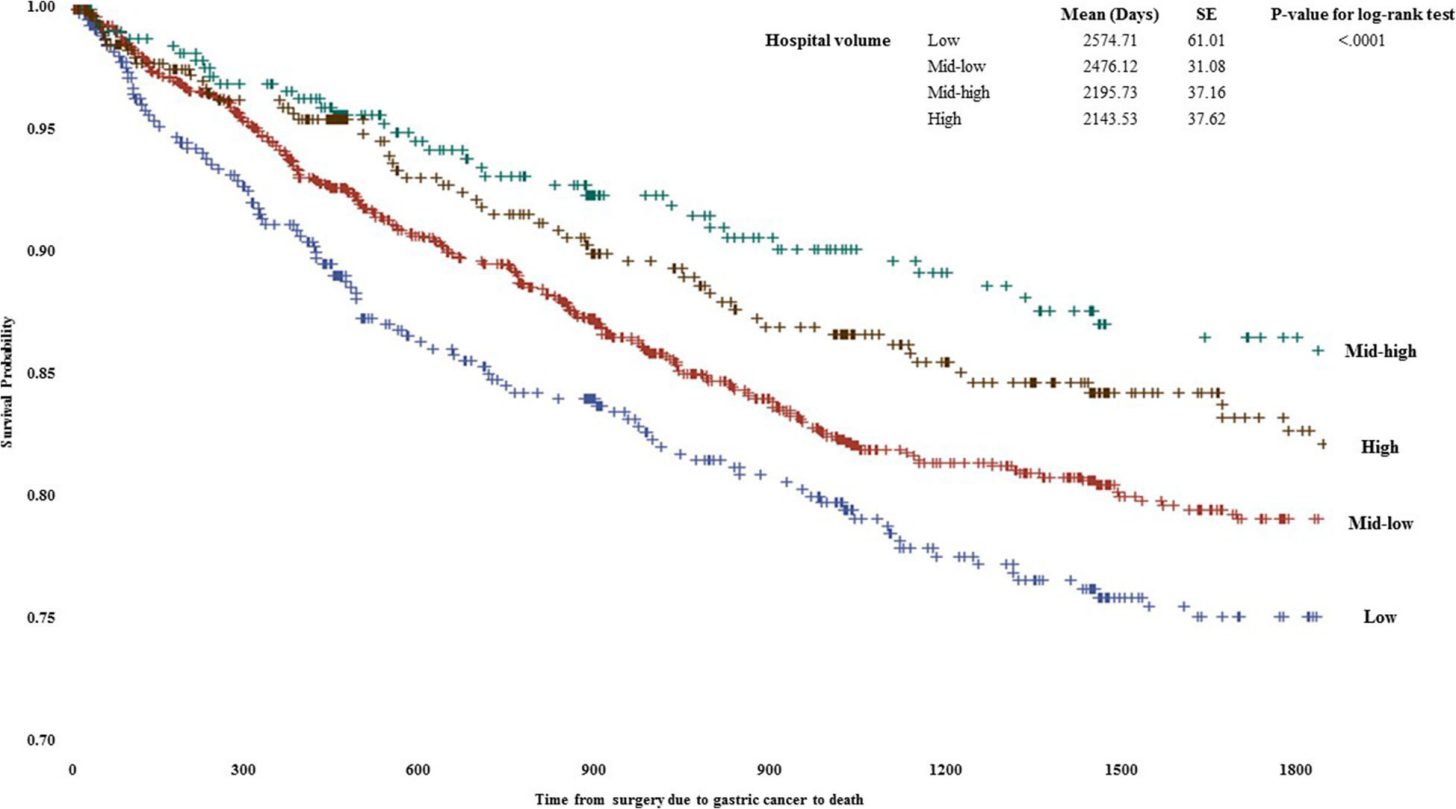
Kaplan-Meier survival curves and log-rank test results comparing survival rates between patients with stomach cancer by surgical volume

Kaplan-Meier survival curves with log-rank test for suggesting cancer-specific survival time by the surgical volume were shown in Fig. 1. The survival time from the first diagnosis of stomach cancer to cancer-specific death was shorter in patients who received surgical treatment at hospitals with lower surgical volumes (*P* for log-rank test <0.0001).

The results of survival analysis to analyze the association between surgical volume and cancer-specific mortality among patients with stomach cancer are shown in Table 2. Although the risk of mortality among patients who received surgical treatment gradually decreased with increasing surgical volume, the mortality risk increased again in “high” surgical volume hospitals, producing a j-shaped curve (mid-low = hazard ratio (HR) 0.773, 95% confidence interval (CI) 0.608–0.983; mid-high = HR 0.541, 95% CI 0.372–0.788; high = HR 0.659, 95% CI 0.473–0.917; ref = low). In addition, the risk of morality from cancer was significantly different according to severity indicators. Patients who received total gastrectomy or complex therapy, including surgery, as treatment for stomach cancer had a higher risk of mortality, although stratifying by CCI was not statistically significant.

**Table 2:** Results of survival analysis using the Cox proportional hazard model to examine the association between surgical volume and cancer-specific mortality

Variables	Mortality due to cancer			
	HR	95% CI		*P* value
Patient characteristics				
Sex				
Male	1.132	0.914	1.401	0.2571
Female	1.000	–	–	–
Age (years)				
−39	1.000	–	–	–
40–49	1.036	0.568	1.892	0.9071
50–59	1.136	0.640	2.016	0.6640
60–69	1.805	1.031	3.162	0.0388
70+	4.252	2.406	7.513	<.0001
Income				
−40	1.033	0.755	1.412	0.8410
41–70	1.166	0.859	1.582	0.3249
71–90	1.077	0.797	1.455	0.6303
91+	1.000	–	–	–
Types of insurance coverage				
Medical Aid	1.393	0.863	2.247	0.1749
NHI, self-employed	0.991	0.808	1.217	0.9338
NHI, employed	1.000	–	–	–
Year of surgery				
2004	1.000	–	–	–
2005	0.881	0.618	1.255	0.4818
2006	0.854	0.589	1.240	0.4078
2007	1.008	0.707	1.438	0.9640
2008	0.765	0.519	1.127	0.1752
2009	1.050	0.714	1.543	0.8043
2010	0.787	0.525	1.179	0.2460
2011	0.664	0.415	1.061	0.0868
2012	1.173	0.733	1.879	0.5060
2013	0.948	0.403	2.232	0.9032
Types of surgery				
Total gastrectomy	2.228	1.823	2.724	<.0001
Subtotal gastrectomy	1.000	–	–	–
Types of treatment				
With chemotherapy and radiotherapy	4.605	3.010	7.043	<.0001
With chemotherapy	6.139	4.935	7.636	<.0001
With radiotherapy	9.068	4.071	20.199	<.0001
Only surgery	1.000	–	–	–
Dissection of lymph node				
Yes	0.983	0.403	2.398	0.9708
No	1.000	–	–	–
CCI	1.051	0.995	1.111	0.0739
Hospital characteristics				
Hospital volume				
Low	1.000	–	–	–
Mid-low	0.773	0.608	0.983	0.0356
Mid-high	0.541	0.372	0.788	0.0014
High	0.659	0.473	0.917	0.0135
Region of hospital				
Capital area	1.131	0.924	1.385	0.2316
Others	1.000	–	–	–
Types of medical institution				
General hospital	0.311	0.133	0.729	0.0072
Hospital	1.000	–	–	–

**Fig. 2:**
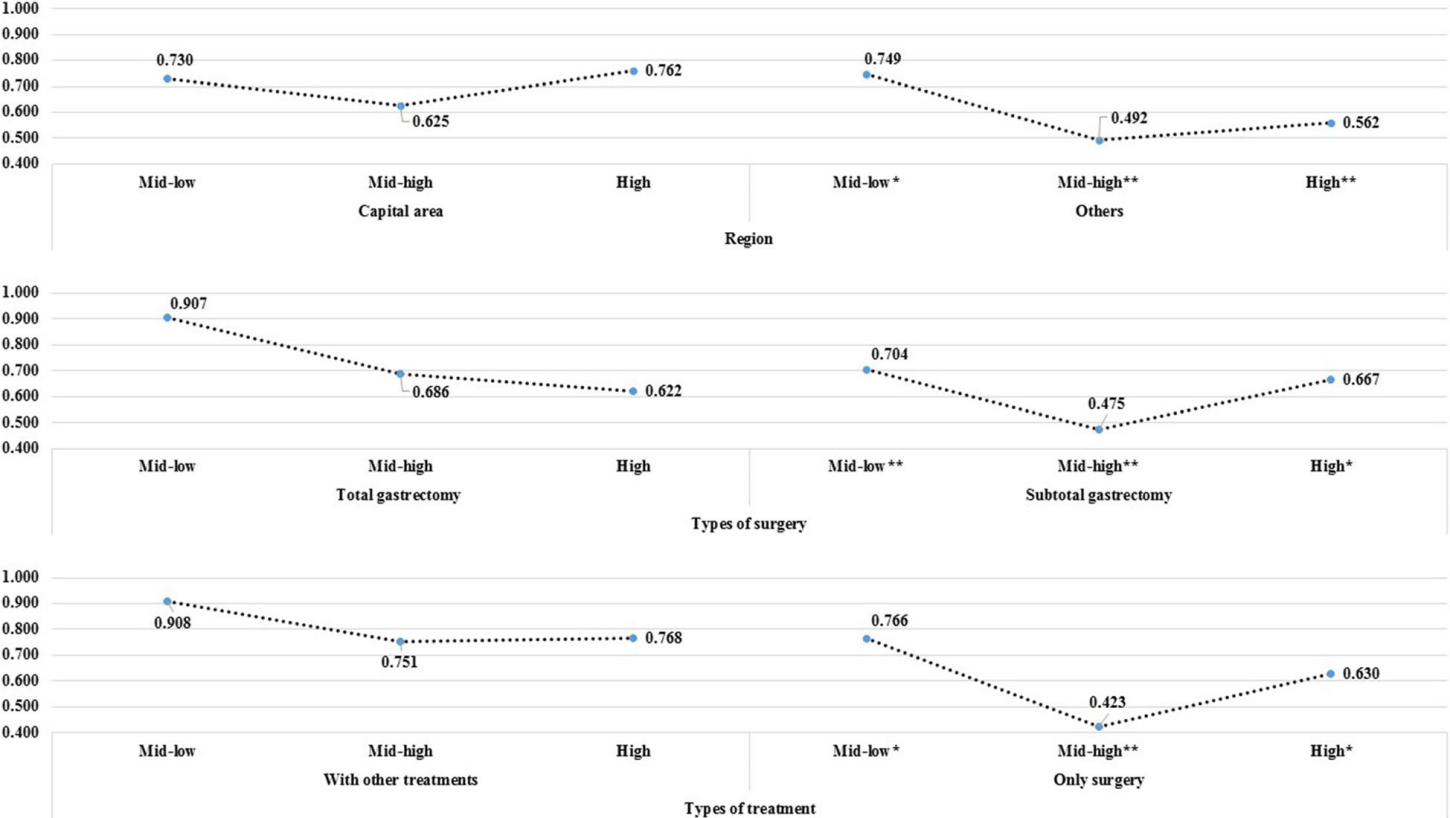
Results of sub-group analyses for the relationship between surgical volume and mortality due to cancer according to hospital regions, types of surgery, or types of treatment. The references level was low in surgical volume groups. *P < 0.1; **P < 0.05

We also performed sub-group analyses to investigate differences in the relationship between surgical volume and cancer-specific mortality according to region and types of surgery or treatment. The risk of mortality gradually decreased with increasing surgical volume, although some j-shaped curve trends were observed. The reduction in mortality risk by higher surgical volume was greater in patients who received surgical treatment at hospitals located outside the capital, as well as in those who received subtotal gastrectomy or only surgery (Fig. 2).

## Discussion

As cancer patients were continuously increased, healthcare professionals have put a significant amount of effort to achieve better surgical outcomes, including the analysis of volume-outcome relationship. According to previous studies, patients who received treatment at hospitals with high surgical volume had better outcomes. Although the authors of those studies suggested alternatives to improve patient outcomes, only a few identified threshold values for the volume-outcome relationship [10, 11]. Therefore, to improve surgical outcomes, we investigated the relationship between surgical volumes that were categorized by the Youden index and cancer-specific mortality among patients with stomach cancer.

The risk of mortality from cancer was lower in hospitals with higher surgical volumes, similar to previous studies on the volume-outcome relationship; however, such positive effects decreased after exceeding a certain level of surgical volume [10, 21]. Our findings may suggest a guideline for maximizing the surgical outcomes of stomach cancer patients. Hospitals with mid-high surgical volumes had the best outcomes. Considering the fact that data in this study were collected by a systematic sampling method (about 2.2% of the entire population), the optimal threshold of surgical volume was approximately 727.3 surgical cases for stomach cancer per hospital annually (16.0 * 100/2.2 = 727.3). However, if surgical volume exceeded about 1363.6, the shown benefit disappeared.

The current study also revealed some interesting findings by sub-group analyses. For instance, reductions in mortality from cancer showed different trends according to region, type of surgery, and type of treatment. The volume-outcome relationship was only statistically significant in hospitals outside the capital region. We hypothesized that there were no specific differences in surgical skills between hospitals in the capital area, since all of the hospitals had substantial surgical volumes for cancer and, therefore, sufficient experience [20, 22]. Differences by the type of surgery or treatment may have resulted as better surgical skills have less impact on severe cases [16, 18]. Therefore, the positive effect of higher surgical volume was only observed for patients undergoing subtotal gastrectomy or only surgery. Based on our results, health policymakers and healthcare professionals may have to figure out alternatives or guidelines that can maximize patient outcomes.

Our study had some strengths. First, our data used a national sampling cohort to investigate the association between surgical volume for stomach cancer and cancer-specific mortality. Thus, the results of this study would be a positive role in making evidence-based cancer policies for optimal management of stomach patients who require surgical treatment. Second, to suggest an optimal threshold for surgical volume, we calculated the cutoff value for surgical volume using the Youden index. This enabled the selection of an optimal threshold for surgical volume with the best outcomes for stomach cancer surgery. Although a number of previous studies investigated surgical volume-outcome relationship in cancer, only a few suggested a threshold volume for efficient outcomes. Therefore, our findings may suggest helpful evidence-based criteria or methods for the cancer policies for optimal management of cancer patients.

Our study also had some limitations. Cancer staging is a major factor in cancer treatment, as it reflects severity of cancer patients. Health outcomes of cancer patients differ based on cancer staging, and a patient’s hospital preference might also differ by their clinical severity. However, we could not identify any information on cancer staging, including the SEER summary staging, due to limitation of data [23]. To minimize such limitation, we considered types of surgery, types of treatment during treatment period of each patient, and dissection of lymph node on surgery as covariates. Second, we only included patients who received surgical treatment for stomach cancer to avoid large differences in severity [2], as healthcare claim data had no information on cancer staging such as SEER. Third, given that the average period from the first diagnosis of stomach cancer to death was about 4 years, there were several potential confounders related to disease management such as detailed severity indicators, socioeconomic factors, or food consumption. All of the listed factors have the potential to influence health outcomes in patients undergoing stomach cancer surgery, even with the inclusion of variables such as the Charlson Comorbidity Index, which reflects the clinical status of patients with gastric cancer [24]. However, since our study used health insurance claim data, which were collected to provide payment for both patients and providers based on medical utilization, we could not account for such confounders. Fourth, detailed characteristics of hospital and human resources, such as surgeon volume or surgeon experience, could serve a positive role in cancer care, particularly for surgical outcomes. However, we could not consider such detailed characteristics due to data limitation, despite consideration of some hospital characteristics. Fifth, the Health Insurance Review and Assessment (HIRA) in South Korea initiated healthcare quality assessments for managing gastric cancer patients after 2014. While positive impacts on quality of care are expected, the current study was conducted before this program was applied. Finally, South Korea is known to offer one of the world’s best gastric cancer care systems, due to its relatively high incidence and the corresponding burden of the disease. Therefore, surgical volumes of gastric cancer may not be generalized in other countries, and healthcare systems and implementation of the recommended thresholds may not be realistic. Nevertheless, suggestion of alternatives in finding the optimal surgical volume for maximizing surgical outcomes can be helpful in establishing an efficient healthcare policy or providing optimal case management for cancer care [25]. Despite some limitations, our findings suggested that cancer mortality decreased in hospitals with higher surgical volumes, as similarly stated in previous studies on the volume-outcome relationship. However, such positive effects decreased for very high-volume hospitals. These associations were especially significant in regions with low surgical volumes and less severe cases. Although further studies using more detailed data are required, our results underscore the need for health policymakers and healthcare professionals to identify effective ways to improve cancer management and reduce stomach cancer mortality in South Korea.

## Conclusion

Higher surgical volume for stomach cancer is associated with lower mortality. Our results suggest that the optimal threshold of surgical volume for good outcomes was about 727.3 surgical cases for stomach cancer per hospital annually in South Korea, but such positive effects decreased after exceeding a certain level of surgeries.

## Abbreviation

CCI: Charlson Comorbidity Index
CI: Confidence interval
HIRA: Health Insurance Review and Assessment
HR: Hazard ratio
ICD: International Classification of Diseases
NHI: National Health Insurance
NHIS: National Health Insurance Service
